# Supplementary material for: Rearrangement of the transmembrane domain interfaces associated with the activation of a GPCR hetero-oligomer
Source: Nat Commun. 2019 Jun 24;10:2765. doi: 10.1038/s41467-019-10834-5 (PMC6591306; doi:10.1038/s41467-019-10834-5)
Supplement: Supplementary file 1 — Supplementary Information [file 41467_2019_10834_MOESM1_ESM.pdf]

Supplementary Information

Rearrangement of the transmembrane domain interfaces associated with the activation of a GPCR hetero-oligomer

Li Xue[1], Qian Sun[1], Han Zhao[1], Xavier Rovira[2,3], Siyu Gai[1], Qianwen He[1], Jean-Philippe Pin[2], Jianfeng Liu[1] and Philippe Rondard[2]

[1] Cellular Signaling laboratory, International Research Center for Sensory Biology and Technology of MOST, Key Laboratory of Molecular Biophysics of MOE, and College of Life Science and Technology, Huazhong University of Science and Technology, Wuhan, Hubei 430074, China.

[2] Institut de Génomique Fonctionnelle (IGF), CNRS, INSERM, Université de Montpellier, Montpellier, France

[3] Present address : Molecular Photopharmacology Research Group, The Tissue Repair and Regeneration Laboratory, University of Vic - Central University of Catalonia, C. de la Laura, 13, 08500 Vic, Spain.

These authors contributed equally : Li Xue, Qian Sun, Han Zhao, Xavier Rovira,

Correspondence and requests for materials should be addressed to J.-P.P (email : jean-philippe.pin@igf.cnrs.fr)
or to J.L. (email: jfliu@mail.hust.edu.cn)

Supplementary Results

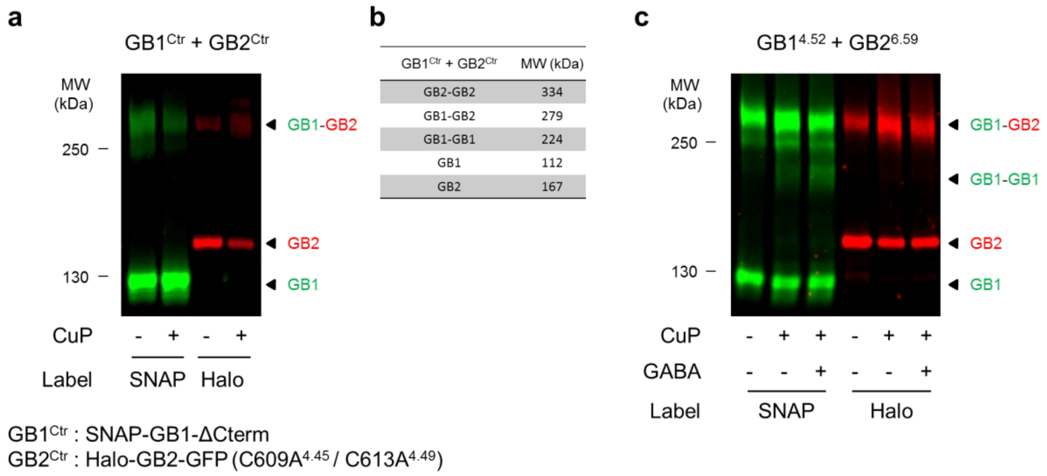

$GB1^{Ctr}$ + $GB2^{Ctr}$	MW (kDa)
GB2-GB2	334
GB1-GB2	279
GB1-GB1	224
GB1	112
GB2	167

Supplementary Figure 1

SDS-PAGE analysis of the GABA$_B$ subunits. (a) Detection of the co-expressed SNAP-tagged GB1^{Ctr} and Halo-tagged GB2^{Ctr}, after labeling with non-cell permeable fluorescent substrates. SNAP-tag and Halo-tag were labeled with SNAP-Alexa Fluor647 and Halo-Alexa Fluor660, respectively. **(b)** Molecular weights (MW) of the indicated species. **(c)** By introducing cysteine mutations in GB1 7TM, for example as indicated here, the cross-linked GB1-GB1 dimers (224 kDa) can be specifically detected.

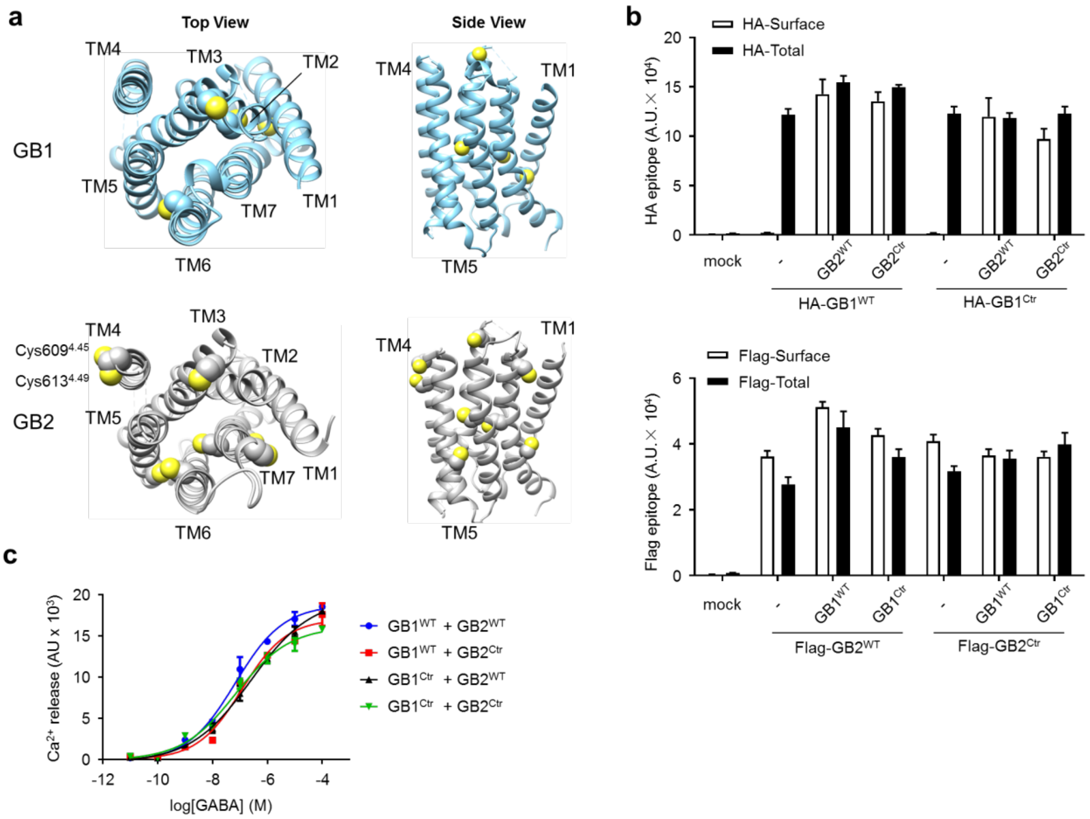

Supplementary Figure 2

Functional analysis of the coexpressed GB1^{Ctr} and GB2^{Ctr} subunits. (a) Location of the 5 and 9 endogenous cysteines (yellow ball) in the TM helices of GB1 (blue) and GB2 (grey), respectively, shown both in top and side views. Two endogenous Cys residues in the GB2^{TM4} that form inter-subunit cross-linking, Cys609$^{4.45}$ and Cys613$^{4.49}$, were mutated to alanine. **(b)** Surface and total cell levels of the indicated HA-tagged GB1 and Flag-tagged GB2 subunits, expressed alone or co-expressed as indicated. ELISA quantification of HA-tagged GB1 or Flag-tagged GB2 at the cell surface (non-permeabilized cells, open bars) and in permeabilized cells (closed bars). Data are mean ± SEM from a typical experiment performed three times. **(c)** Intracellular Ca^{2+} response upon activation by GABA, of the indicated GB1 subunit co-expressed with the indicated GB2 constructs. Data are mean ± SEM from a typical experiment performed three times. Of note, the GB1^{Ctr} and GB2^{Ctr} subunits have similar cell surface targeting and functional properties to the wild-type subunits.

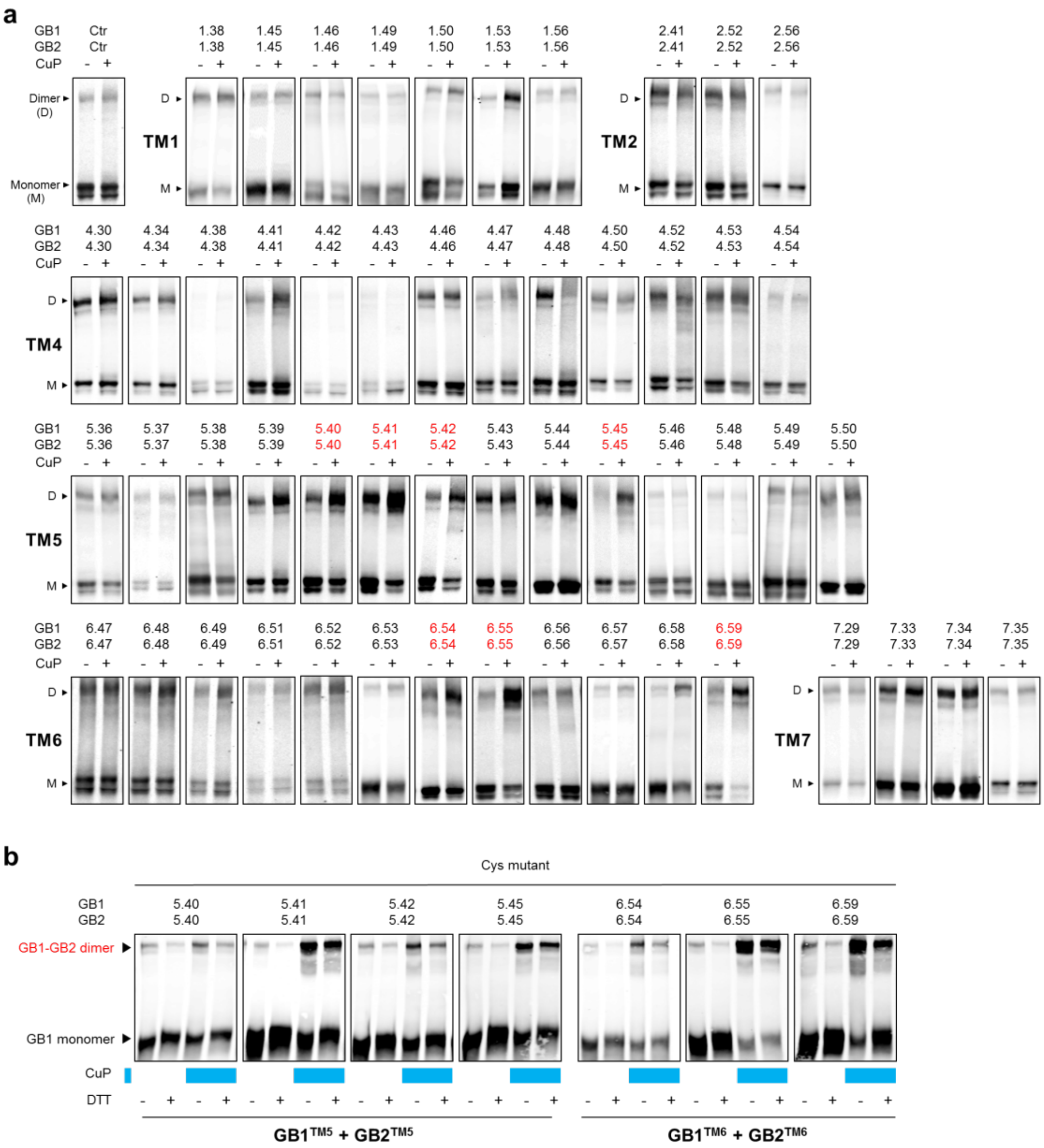

Supplementary Figure 3

Cross-linking of the coexpressed GB1 and GB2 subunits in basal conditions. (a) Cross-linking of the indicated cell surface SNAP-GB1 subunits labeled with fluorescent SNAP substrates, after treatment (+) or without treatment (-) with CuP. After SDS-PAGE in non-reducing conditions, GB1 monomers and GB1-GB2 dimers were detected via the fluorophore covalently attached to the receptors. These blots are from a typical experiment performed at least three times. Of note, the constructs highlighted in red are those that produced a large increase of the ratio between GB1-GB2 dimer over the total of GB1 subunit after CuP treatment (see Figure 2d). **(b)** Effect of the indicated reducing agent on the GB1-GB2 dimer band.

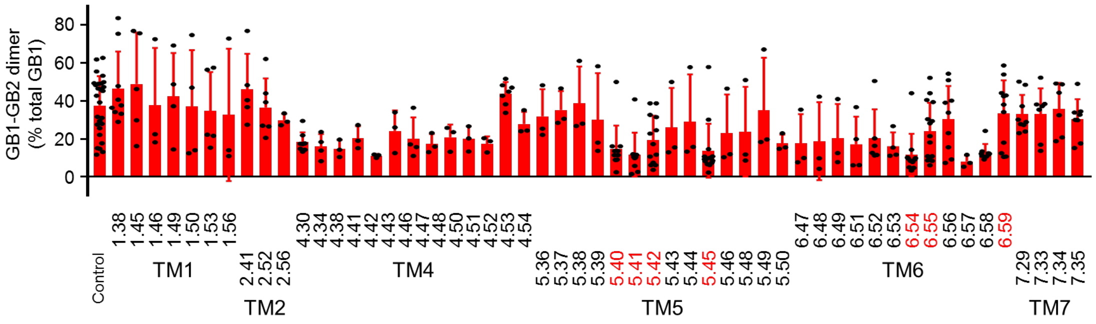

Supplementary Figure 4

Ratio between GB1-GB2 dimer over the total amount of SNAP-tagged GB1 signal quantified from blots, under basal condition in the absence of CuP treatment. Data are mean ± SD from at least three independent experiments (n = 3-22). Of note, high variability probably results from differences in the expression level and in sample preparation between experiments.

TM1			TM2			TM4			TM5			TM6			TM7		
	GB1	GB2		GB1	GB2		GB1	GB2		GB1	GB2		GB1	GB2		GB1	GB2
1.38	K589C	P479C	2.41	L631C	L521C	4.30	W707C	Q594C	5.36	M765C	M652C	6.46	V811C	-	7.29	F833C	F720C
1.45	V596C	A486C	2.45	G635C	G525C	4.34	A711C	V598C	5.37	N766C	T653C	6.47	A812C	G699C	7.30	A834C	-
1.46	L597C	L487C	2.52	A642C	S532C	4.38	L715C	G602C	5.38	T767C	I654C	6.48	V813C	I700C	7.33	S837C	A724C
1.49	L600C	L490C	2.56	L646C	F536C	4.41	G718C	L605C	5.39	W768C	W655C	6.49	L814C	M701C	7.34	L838C	L725C
1.50	G601C	G491C	2.59	D649C	D539C	4.42	M719C	I606C	5.40	L769C	L656C	6.51	L816C	I703C	7.35	A839C	V726C
1.53	L604C	M494C				4.43	D720C	D607C	5.41	G770C	G657C	6.52	I817C	I704C			
1.56	V607C	A497C				4.44	V721C	L608C	5.42	I771C	I658C	6.53	T818C	G705C			
						4.45	L722C	C609A	5.43	F772C	V659C	6.54	A819C	A706C			
						4.46	T723C	I610C	5.44	Y773C	Y660C	6.55	P820C	A707C			
						4.47	L724C	L611C	5.45	G774C	A661C	6.56	V821C	V708C			
						4.48	A725C	I612C	5.46	Y775C	Y662	6.57	T822C	S709C			
						4.49	I726C	C613A	5.47	K776C	K663C	6.58	M823C	F710C			
						4.50	W727C	W614C	5.48	G777C	G664C	6.59	I824C	L711C			
						4.51	Q728C	Q615C	5.49	L778C	L665C						
						4.52	I729C	A616C	5.50	L779C	L666C						
						4.53	V730C	V617C									
						4.54	D731C	D618C									
						4.55	P732C	P619C									

Supplementary Figure 5

GB1 and GB2 mutants in which the indicated single mutation was introduced in the GB1^{Ctr} and GB2^{Ctr} subunits, respectively, in the different TM domains indicated. The positions where the introduction of a Cys in both GB1 and GB2 subunits led to efficient cross-linking between GB1 and GB2 (see Supplementary Figure 3a) are highlighted in red.

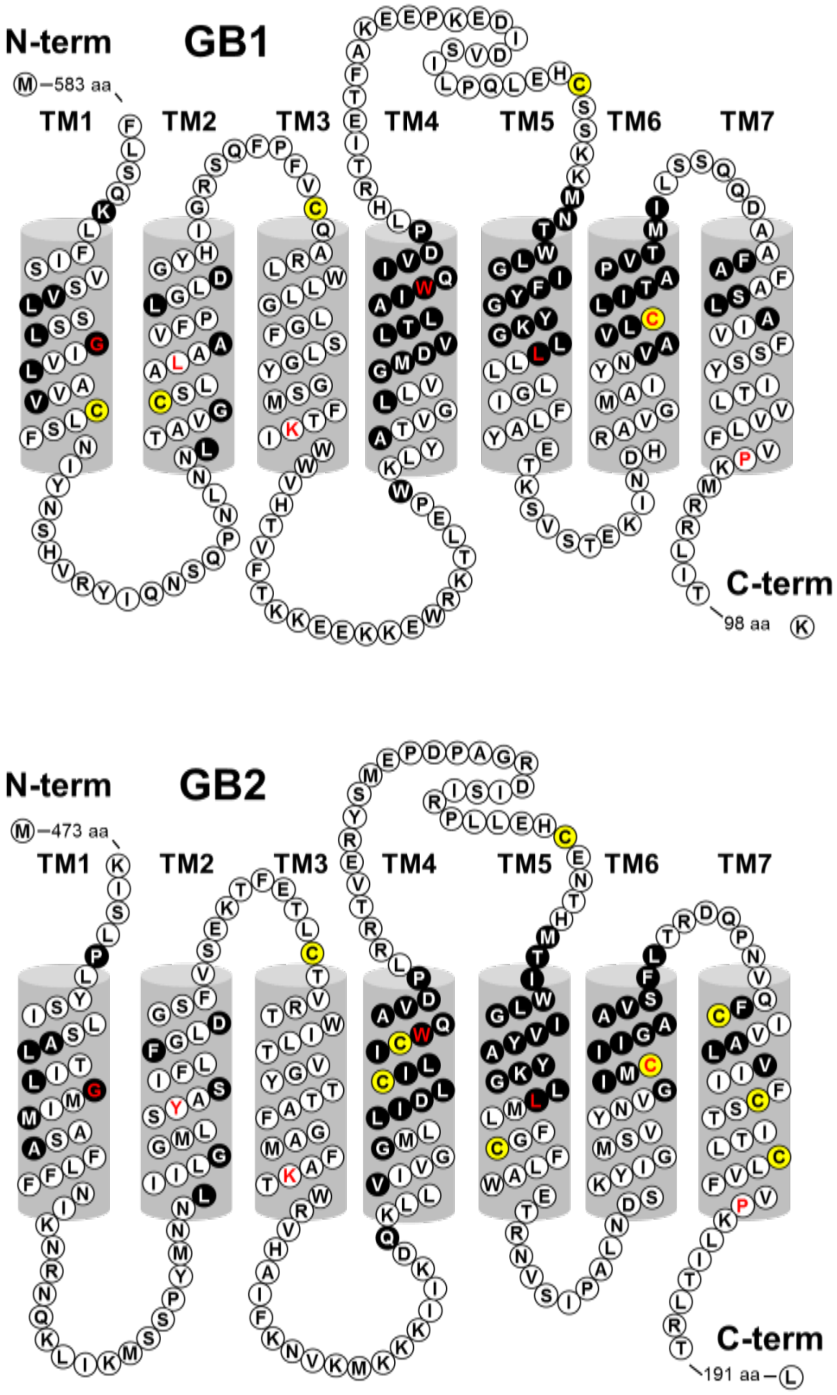

Supplementary Figure 6

Secondary structure of the 7TM domain of the rat wild-type GB1 and GB2 subunits. Amino acids that were replaced with cysteine residues are highlighted by black circles. The endogenous cysteine residues are highlighted in yellow. The most conserved residues in each TM domain (X.50) are highlighted in red.

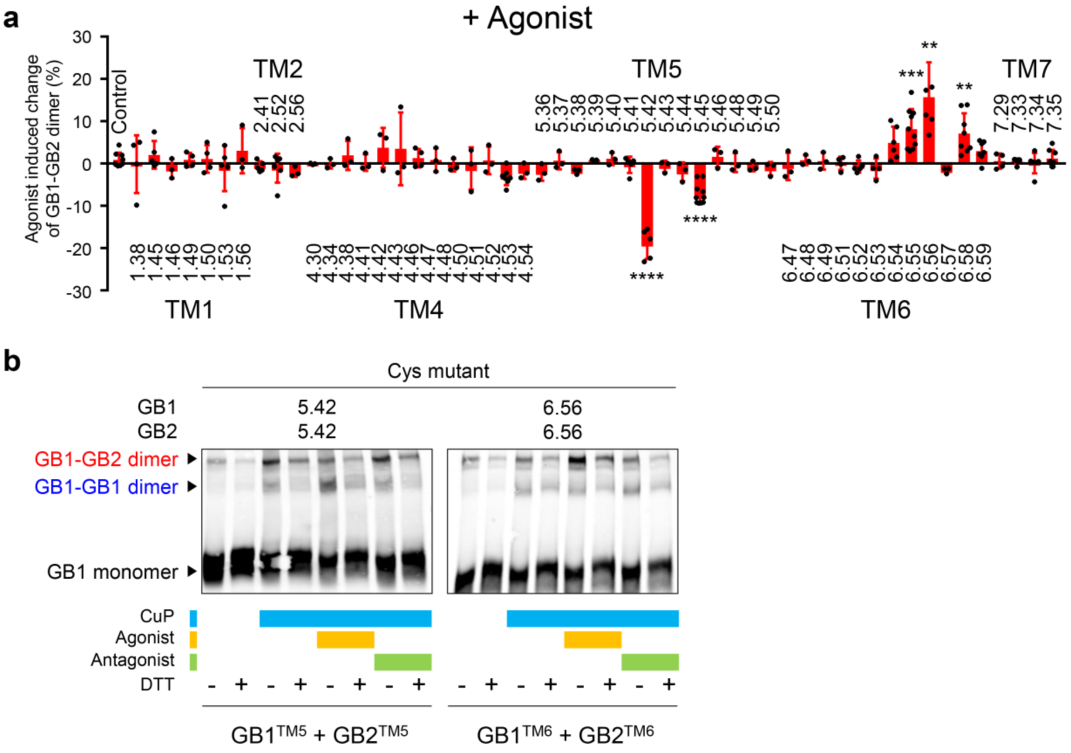

Supplementary Figure 7

(a) Change of GB1-GB2 dimer cross-linking induced by the agonist and determined by GB1-GB2 dimer quantification before and after GABA treatment. **(b)** Effect of the indicated reducing agent on the GB1-GB2 dimer or GB1-GB1 dimer bands.

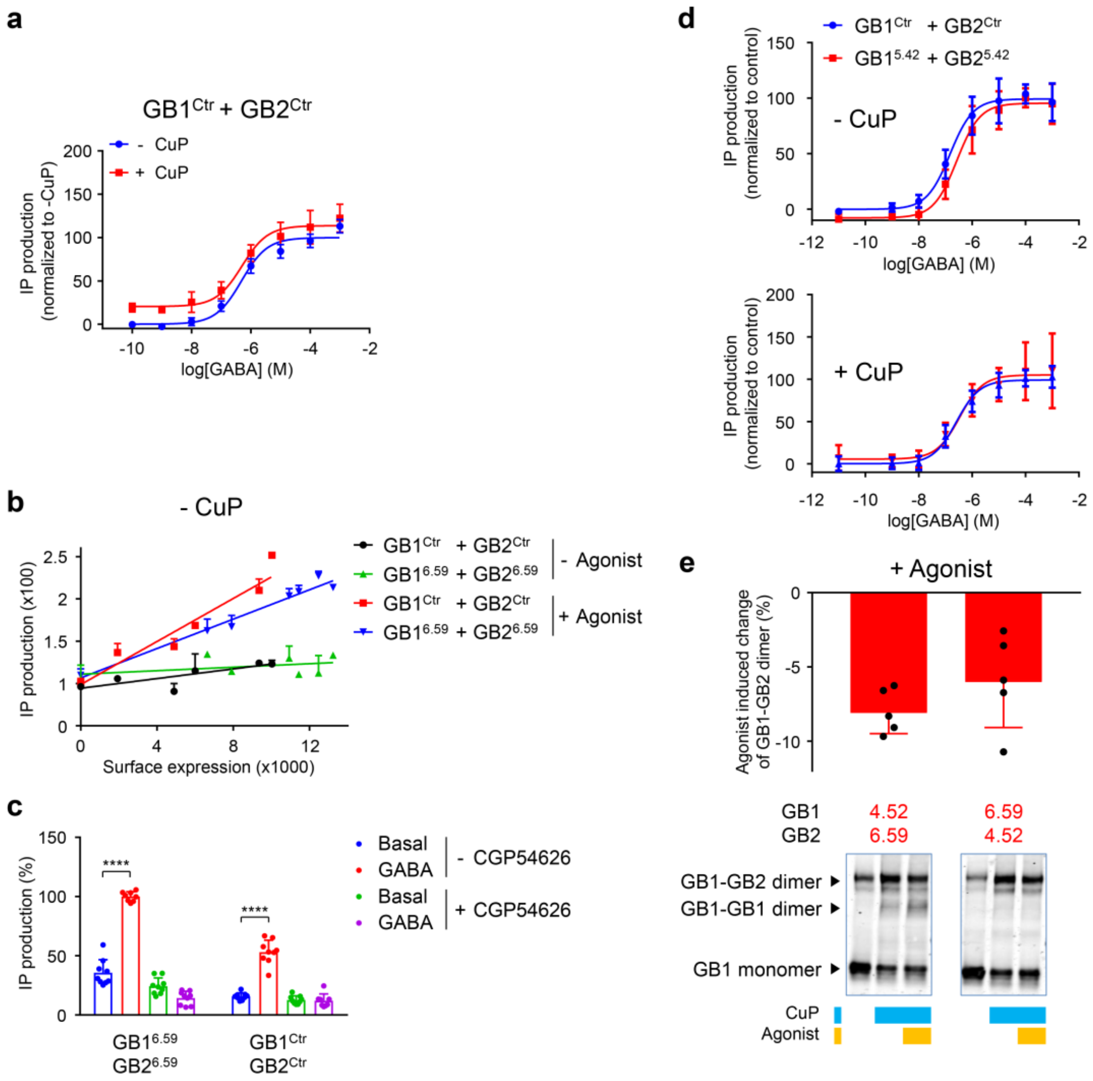

Supplementary Figure 8

(a) IP production in cells that co-express the GB1^{Ctr} and GB2^{Ctr} subunits after treatment with or without CuP and stimulation with GABA. **(b)** In the absence of CuP treatment, both the co-expressed GB1^{Ctr} and GB2^{Ctr} subunits and the co-expressed mutants GB1$^{6.59}$ and GB2$^{6.59}$ have similar properties to induce IP production both in basal conditions and after stimulation with GABA. Data are mean ± SD from a typical experiment performed three times. **(c)** Similar experiments in the absence of CuP, but with or without competitive antagonist treatment. Data are mean ± SD from three independent experiments. **(d)** IP production in cells that co-express the GB1$^{5.42}$ and GB2$^{5.42}$ subunits after treatment with or without CuP and stimulation with GABA. **(e)** Change of GB1-GB2 dimer rate over the total GB1 subunit induced by the agonist and determined by the GB1-GB2 dimer rate quantification before and after GABA treatment. Blots are representative of a typical experiment performed three times.

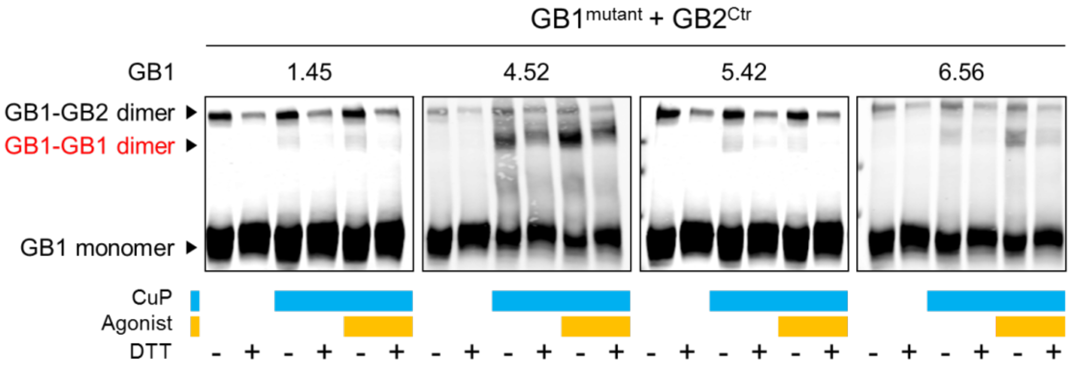

Supplementary Figure 9

Effect of the indicated reducing agent on the GB1-GB1 dimer band.

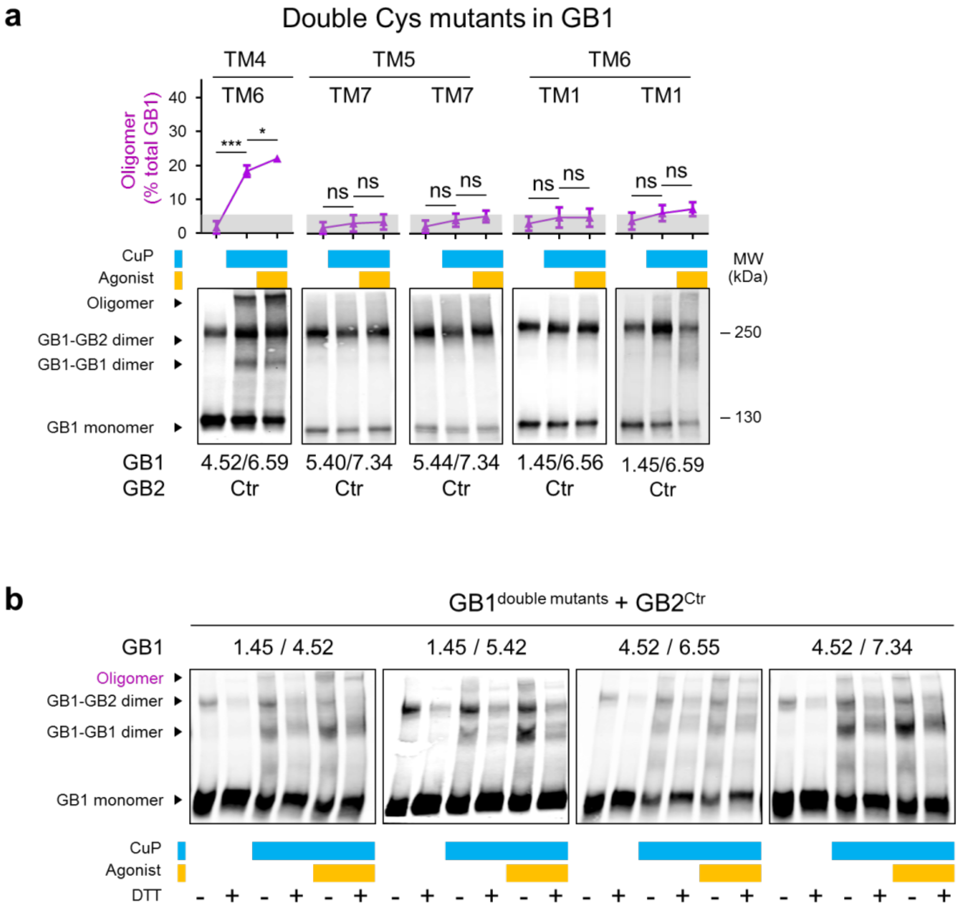

Supplementary Figure 10

(a) Quantification of the high-molecular-weight complexes (oligomers) obtained after cross-linking of double cysteine substitutions in different TMs of the GB1 subunit, after pre-incubation, or not, with GABA, and with CuP, as indicated. The percentage of oligomers (in purple) relative to the total amount of GB1 subunit was quantified by imaging the fluorescent blots. Data are mean ± SD of at least three individual experiments (n = 3-5). **(b)** Effect of the indicated reducing agent on the oligomer band.

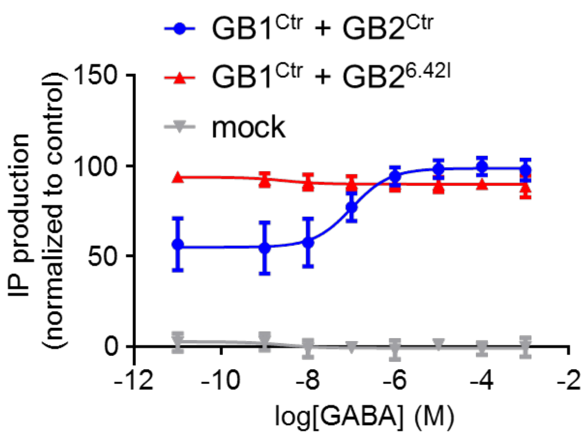

Supplementary Figure 11

GABA dose-response curve of IP production in cells that co-express the $GB1^{Ctr}$ with $GB2^{Ctr}$ or $GB2^{6.42I}$ subunits.

HA-SNAP-GB1a

EcoRI	ATG	mGlu5 SP	HA	MluI	SNAP	MluI	GB1a	XbaI
		TCCTTCTGTTG	TACCCCT	ACGCGT	GGATCCGACAAAGACTGCGAAATGAAGCGCACCACCCTGGATAGCCCTCTGGGCAAGCTGGAACTGTCTGGGTGCGAACAGGGC	ACGCGT		
		ATCCTGTCAG	ACGACG		CTGCACGAGATCAAGCTGCTGGGCAAAGGAACATCTGCCGCCGACGCCGTGGAAGTGCCTGCCCCAGCCGCCGTGCTGGGCGGA			
		TCCTACTTCTG	TACCCG		CCAGAGCCACTGATGCAGGCCACCGCCTGGCTCAACGCCTACTTTCACCAGCCTGAGGCCATCGAGGAGTTCCCTGTGCCAGCCCT			
		AAAGAAGAT	ACTACG		GCACCACCCAGTGTTCCAGCAGGAGAGCTTTACCCGCCAGGTGCTGTGGAAACTGCTGAAAGTGGTGAAGTTCGGAGAGGTCATC			
		GTACGAGGG	CC		AGCTACCAGCAGCTGGCCGCCCTGGCCGGCAATCCCGCCGCCACCGCCGCCGTGAAAACCGCCCTGAGCGGAAATCCCGTGCCCA			
		AGTGCACAGT			TTCTGATCCCCTGCCACCGGGTGGTGTCTAGCTCTGGCGCCGTGGGGGGCTACGAGGGCGGGCTCGCCGTGAAAGAGTGGCTGCT			
		CCACGCGA			GGCCCACGAGGGCCACAGACTGGGCAAGCCTGGGCTGGGCGGATCCGACAAAGACTGCGAAATGAAGCGCACCACCCTGGATA			
					GCCCTCTGGGCAAGCTGGAACTGTCTGGGTGCGAACAGGGCCTGCACGAGATCAAGCTGCTGGGCAAAGGAACATCTGCCGCCG			
					ACGCCGTGGAAGTGCCTGCCCCAGCCGCCGTGCTGGGCGGACCAGAGCCACTGATGCAGGCCACCGCCTGGCTCAACGCCTACTT			
					TCACCAGCCTGAGGCCATCGAGGAGTTCCCTGTGCCAGCCCTGCACCACCCAGTGTTCCAGCAGGAGAGCTTTACCCGCCAGGTGC			
					TGTGGAAACTGCTGAAAGTGGTGAAGTTCGGAGAGGTCATCAGCTACCAGCAGCTGGCCGCCCTGGCCGGCAATCCCGCCGCCAC			
					CGCCGCCGTGAAAACCGCCCTGAGCGGAAATCCCGTGCCCATTCTGATCCCCTGCCACCGGGTGGTGTCTAGCTCTGGCGCCGTGG			
					GGGGCTACGAGGGCGGGCTCGCCGTGAAAGAGTGGCTGCTGGCCCACGAGGGCCACAGACTGGGCAAGCCTGGGCTGGGCGGA			
					TCCGACAAAGACTGCGAAATGAAGCGCACCACCCTGGATAGCCCTCTGGGCAAGCTGGAACTGTCTGGGTGCGAACAGGGCCTGC			
					ACGAGATCAAGCTGCTGGGCAAAGGAACATCTGCCGCCGACGCCGTGGAAGTGCCTGCCCCAGCCGCCGTGCTGGGCGGACCAG			
					AGCCACTGATGCAGGCCACCGCCTGGCTCAACGCCTACTTTCACCAGCCTGAGGCCATCGAGGAGTTCCCTGTGCCAGCCCTGCAC			
					CACCCAGTGTTCCAGCAGGAGAGCTTTACCCGCCAGGTGCTGTGGAAACTGCTGAAAGTGGTGAAGTTCGGAGAGGTCATCAGCT			
					ACCAGCAGCTGGCCGCCCTGGCCGGCAATCCCGCCGCCACCGCCGCCGTGAAAACCGCCCTGAGCGGAAATCCCGTGCCCATTCT			
					GATCCCCTGCCACCGGGTGGTGTCTAGCTCTGGCGCCGTGGGGGGCTACGAGGGCGGGCTCGCCGTGAAAGAGTGGCTGCTGGC			
					CCACGAGGGCCACAGACTGGGCAAGCCTGGGCTGGGCGGATCCGACAAAGACTGCGAAATGAAGCGCACCACCCTGGATAGCCC			
					TCTGGGCAAGCTGGAACTGTCTGGGTGCGAACAGGGCCTGCACGAGATCAAGCTGCTGGGCAAAGGAACATCTGCCGCCGACGC			
					CGTGGAAGTGCCTGCCCCAGCCGCCGTGCTGGGCGGACCAGAGCCACTGATGCAGGCCACCGCCTGGCTCAACGCCTACTTTCAC			
					CAGCCTGAGGCCATCGAGGAGTTCCCTGTGCCAGCCCTGCACCACCCAGTGTTCCAGCAGGAGAGCTTTACCCGCCAGGTGCTGTG			
					GAAACTGCTGAAAGTGGTGAAGTTCGGAGAGGTCATCAGCTACCAGCAGCTGGCCGCCCTGGCCGGCAATCCCGCCGCCACCGCC			
					GCCGTGAAAACCGCCCTGAGCGGAAATCCCGTGCCCATTCTGATCCCCTGCCACCGGGTGGTGTCTAGCTCTGGCGCCGTGGGGG			
					GCTACGAGGGCGGGCTCGCCGTGAAAGAGTGGCTGCTGGCCCACGAGGGCCACAGACTGGGCAAGCCTGGGCTGGGC			

FLAG-HALO-GB2

HindIII	ATG	CD8a SP	FLAG	ClaI	HALO	EcoRV	GB2	XbaI
		GCCTTACCAG	GACTAC	ATCGAT	GCAGAAATCGGTACTGGCTTTCCATTCGACCCCCATTATGTGGAAGTCCTGGGCGAGCGCATGCACTACGTCGATGTTGGTCCGCG	GATATC		
		TGACCGCCTT	AAGGAC		CGATGGCACCCCTGTGCTGTTCCTGCACGGTAACCCGACCTCCTCCTACGTGTGGCGCAACATCATCCCGCATGTTGCACCGACCCA			
		GCTCCTGCCG	GACGAT		TCGCTGCATTGCTCCAGACCTGATCGGTATGGGCAAATCCGACAAACCAGACCTGGGTTATTTCTTCGACGACCACGTCCGCTTCAT			
		CTGGCCTTGC	GACAAG		GGATGCCTTCATCGAAGCCCTGGGTCTGGAAGAGGTCGTCCTGGTCATTCACGACTGGGGCTCCGCTCTGGGTTTCCACTGGGCCA			
		TGCTCCACGC	GCCGGC		AGCGCAATCCAGAGCGCGTCAAAGGTATTGCATTTATGGAGTTCATCCGCCCTATCCCGACCTGGGACGAATGGCCAGAATTTGCC			
		CGCCAGGCCG			CGCGAGACCTTCCAGGCCTTCCGCACCACCGACGTCGGCCGCAAGCTGATCATCGATCAGAACGTTTTTATCGAGGGTACGCTGCC			
		GCCGCCGCTA			GATGGGTGTCGTCCGCCCGCTGACTGAAGTCGAGATGGACCATTACCGCGAGCCGTTCCTGAATCCTGTTGACCGCGAGCCACTGT			
		GCGGCATC			GGCGCTTCCCAAACGAGCTGCCAATCGCCGGTGAGCCAGCGAACATCGTCGCGCTGGTCGAAGAATACATGGACTGGCTGCACCA			
					GTCCCCTGTCCCGAAGCTGCTGTTCTGGGGCACCCCAGGCGTTCTGATCCCACCGGCCGAAGCCGCTCGCCTGGCCAAAAGCCTGC			
					CTAACTGCAAGGCTGTGGACATCGGCCCGGGTCTGAATCTGCTGCAAGAAGACAACCCGGACCTGATCGGCAGCGAGATCGCGC			
					GCTGGCTGTCGACGCTCGAGATTTCCGGC			

Supplementary Figure 12

Nucleotide sequences of the indicated signal peptide (SP), epitope tag (HA or FLAG), and suicide enzyme tag (SNAP or HALO) for the GB1 and GB2 constructs.

$GABA_{B1}$ mutations

Mutation	Primer name	Primer sequence	Mutation	Primer name	Primer sequence
K589C	XL-GB1-#01	CGTTTCCTGTCTCAGTGTCTCTTTATCTCCGTC	T767C	G1-T767C-F	CTCCAAGAAGATGAATTGCTGGCTTGGCATTTTC
	XL-GB1-#02	GACGGAGATAAAGAGACACTGAGACAGGAAACG		G1-T767C-R	GAAAATGCCAAGCCAGCAATTCATCTTCTTGGAG
V596C	XL-GB1-#27	CTTTATCTCCGTCTCATGTCTCTCCAGCCTGGGC	W768C	XL-GB1-#13	CAAGAAGATGAATACGTGTCTTGGCATTTTCTATG
	XL-GB1-#28	GCCCAGGCTGGAGAGACATGAGACGGAGATAAAG		XL-GB1-#14	CATAGAAAATGCCAAGACACGTATTCATCTTCTTG
L597C	XL-GB1-#29	CTCCGTCTCAGTTTGCTCCAGCCTGGGCATTG	L769C	XL-GB1-#15	GAAGATGAATACGTGGTGTGGCATTTTCTATGG
	XL-GB1-#30	CAATGCCCAGGCTGGAGCAAACTGAGACGGAG		XL-GB1-#16	CCATAGAAAATGCCACACCACGTATTCATCTTC
L600C	XL-GB1-#31	CTCAGTTCTCTCCAGCTGCGGCATTGTTCTTGC	G770C	XL-GB1-#39	GATGAATACGTGGCTTTGCATTTTCTATGGTTAC
	XL-GB1-#32	GCAAGAACAATGCCGCAGCTGGAGAGAACTGAG		XL-GB1-#40	GTAACCATAGAAAATGCAAAGCCACGTATTCATC
G601C	XL-GB1-#57	GTTCTCTCCAGCCTGTGCATTGTTCTTGCTGTTG	I771C	XL-GB1-#41	GAATACGTGGCTTGGCTGTTTCTATGGTTACAAG
	XL-GB1-#58	CAACAGCAAGAACAATGCACAGGCTGGAGAGAAC		XL-GB1-#42	CTTGTAACCATAGAAACAGCCAAGCCACGTATTC
L604C	XL-GB1-#59	CAGCCTGGGCATTGTTTGTGCTGTTGTCTGTCTG	F772C	G1-F772C-F	CGTGGCTTGGCATTTGCTATGGTTACAAGGG
	XL-GB1-#60	CAGACAGACAACAGCACAAACAATGCCCAGGCTG		G1-F772C-R	CCCTTGTAACCATAGCAAATGCCAAGCCACG
V607C	XL-GB1-#61	CATTGTTCTTGCTGTTTGCTGTCTGTCCTTTAAC	Y773C	XL-GB1-#71	GTGGCTTGGCATTTTCTGTGGTTACAAGGGGCTGC
	XL-GB1-#62	GTTAAAGGACAGACAGCAAACAGCAAGAACAATG		XL-GB1-#72	GCAGCCCCTTGTAACCACAGAAAATGCCAAGCCAC
L631C	RG1L631C-F	CCAACCTGAACAATTGCACTGCTGTGGGCTG	G774C	G1-G774C-F	CTTGGCATTTTCTATTGTTACAAGGGGCTGC
	RG1L631C-R	CAGCCCACAGCAGTGCAATTGTTCAGGTTGG		G1-G774C-R	GCAGCCCCTTGTAACAATAGAAAATGCCAAG
G635C	RG1G635C-F	CAATCTGACTGCTGTGTGCTGCTCACTGGCAC	Y775C	XL-GB1-#19	GGCATTTTCTATGGTTGCAAGGGGCTGCTGCTG
	RG1G635C-R	GTGCCAGTGAGCAGCACACAGCAGTCAGATTG		XL-GB1-#20	CAGCAGCAGCCCCTTGCAACCATAGAAAATGCC
A642C	RG1A642C-F	CACTGGCACTGGCTTGTGTCTTCCCTCTCG	K776C	G1-K776C-F	CATTTTCTATGGTTACTGCGGGCTGCTGCTGCTG
	RG1A642C-R	CGAGAGGGAAGACACAAGCCAGTGCCAGTG		G1-K776C-R	CAGCAGCAGCAGCCCGCAGTAACCATAGAAAATG
L646C	RG1L646C-F	GCTGCTGTCTTCCCTTGCGGGCTGGATGGTTAC	G777C	G1-G777C-F	CTATGGTTACAAGTGTCTGCTGCTGCTGCTG
	RG1L646C-R	GTAACCATCCAGCCCGCAAGGGAAGACAGCAGC		G1-G777C-R	CAGCAGCAGCAGCAGACACTTGTAACCATAG
D649C	RG1D649C-F	CCCTCTCGGGCTGTGTGGTTACCACATAG	L778C	G1-L778C-F	CTATGGTTACAAGGGGTGTCTGCTGCTGCTGGGA
	RG1D649C-R	CTATGTGGTAACCACACAGCCCGAGAGGG		G1-L778C-R	TCCCAGCAGCAGCAGACACCCCTTGTAACCATAG
W707C	XL-GB1-#63	GAAGACCCTAGAGCCCTGTAAACTCTATGCCACTG	L779C	XL-GB1-#73	GGTTACAAGGGGCTGTGTCTGCTGCTGGGAATC
	XL-GB1-#64	CAGTGGCATAGAGTTTACAGGGCTCTAGGGTCTTC		XL-GB1-#74	GATTCCCAGCAGCAGACACAGCCCCTTGTAACC
A711C	XL-GB1-#65	CCCTGGAAACTCTATTGCACTGTGGGCCTGCTG	V811C	G1-V811C-F	CATGGCTATCTACAATTGCGCGGTCCTGTGTCTC
	XL-GB1-#66	CAGCAGGCCCACAGTGCAATAGAGTTTCCAGGG		G1-V811C-R	GAGACACAGGACCGCGCAATTGTAGATAGCCATG
L715C	XL-GB1-#67	CTATGCCACTGTGGGCTGTCTGGTGGGCATGGATG	A812C	G1-A812C-F	GCTATCTACAATGTCTGTGTCCTGTGTCTCATC
	XL-GB1-#68	CATCCATGCCCACCAGACAGCCCACAGTGGCATAG		G1-A812C-R	GATGAGACACAGGACACAGACATTGTAGATAGC
G718C	Sun-G718-F	GTGGGCCTGCTGGTGTGCATGGATGTCCTGACT	V813C	G1-V813C-F	CTATCTACAATGTCGCGTGCCTGTGTCTCATCAC
	Sun-G718-R	AGTCAGGACATCCATGCACACCAGCAGGCCCAC		G1-V813C-R	GTGATGAGACACAGGCACGCGACATTGTAGATAG
M719C	Sun-M719-F	GCCTGCTGGTGGGCTGTGATGTCCTGACTCT	L814C	G1-L814C-F	CTACAATGTCGCGGTCTGTTGTCTCATCACTGCTC
	Sun-M719-R	AGAGTCAGGACATCACAGCCCACCAGCAGGC		G1-L814C-R	GAGCAGTGATGAGACAACAGACCGCGACATTGTAG
D720C	Sun-D720-F	CTGCTGGTGGGCATGTGTGTCCTGACTCTTGCC	L816C	XL-GB1-#49	GTCGCGGTCCTGTGTTGCATCACTGCTCCTGTG
	Sun-D720-R	GGCAAGAGTCAGGACACACATGCCCACCAGCAG		XL-GB1-#50	CACAGGAGCAGTGATGCAACACAGGACCGCGAC
V721C	Sun-V721-F	CTGGTGGGCATGGATTGCCTGACTCTTGCCATC	I817C	G1-I817C-F	GCGGTCCTGTGTCTCTGCACTGCTCCTGTGACC
	Sun-V721-R	GATGGCAAGAGTCAGGCAATCCATGCCCACCAG		G1-I817C-R	GGTCACAGGAGCAGTGCAGAGACACAGGACCGC
L722C	XL-GB1-#03	GTGGGCATGGATGTCTGTACTCTTGCCATCTGG	T818C	G1-T818C-F	GTCCTGTGTCTCATCTGTGCTCCTGTGACCATG
	XL-GB1-#04	CCAGATGGCAAGAGTACAGACATCCATGCCCAC		G1-T818C-R	CATGGTCACAGGAGCACAGATGAGACACAGGAC
T723C	Sun-T723-F	GGCATGGATGTCCTGTGTCTTGCCATCTGGCAG	A819C	XL-GB1-#51	CTGTGTCTCATCACTTGTCCTGTGACCATGATC
	Sun-T723-R	CTGCCAGATGGCAAGACACAGGACATCCATGCC		XL-GB1-#52	GATCATGGTCACAGGACAAGTGATGAGACACAG
L724C	Sun-L724-F	CATGGATGTCCTGACTTGTGCCATCTGGCAGATTG	P820C	XL-GB1-#21	GTGTCTCATCACTGCTTGTGTGACCATGATCCTTTC
	Sun-L724-R	CAATCTGCCAGATGGCACAAGTCAGGACATCCATG		XL-GB1-#22	GAAAGGATCATGGTCACACAAGCAGTGATGAGACAC
A725C	XL-GB1-#05	GATGTCCTGACTCTTTGCATCTGGCAGATTGTG	V821C	XL-GB1-#23	CTCATCACTGCTCCTTGTACCATGATCCTTTCC
	XL-GB1-#06	CACAATCTGCCAGATGCAAAGAGTCAGGACATC		XL-GB1-#24	GGAAAGGATCATGGTACAAGGAGCAGTGATGAG
I726C	XL-GB1-#07	GTCCTGACTCTTGCCTGCTGGCAGATTGTGGAC	T822C	XL-GB1-#53	CATCACTGCTCCTGTGTGCATGATCCTTTCCAGTC
	XL-GB1-#08	GTCCACAATCTGCCAGCAGGCAAGAGTCAGGAC		XL-GB1-#54	GACTGGAAAGGATCATGCACACAGGAGCAGTGATG
W727C	Sun-W727-F	CTGACTCTTGCCATCTGTCAGATTGTGGACCCC	M823C	XL-GB1-#55	CACTGCTCCTGTGACCTGTATCCTTTCCAGTCAGC
	Sun-W727-R	GGGGTCCACAATCTGACAGATGGCAAGAGTCAG		XL-GB1-#56	GCTGACTGGAAAGGATACAGGTCACAGGAGCAGTG
Q728C	XL-GB1-#09	CTCTTGCCATCTGGTGTATTGTGGACCCCTTG	I824C	XL-GB1-#25	GCTCCTGTGACCATGTGCCTTTCCAGTCAGCAG
	XL-GB1-#10	CAAGGGGTCCACAATACACCAGATGGCAAGAG		XL-GB1-#26	CTGCTGACTGGAAAGGCACATGGTCACAGGAGC
I729C	XL-GB1-#33	CTTGCCATCTGGCAGTGTGTGGACCCCTTGCAC	F833C	XL-GB1-#43	CAGCAGGACGCAGCCTGTGCCTTTGCCTCTCTG
	XL-GB1-#34	GTGCAAGGGGTCCACACACTGCCAGATGGCAAG		XL-GB1-#44	CAGAGAGGCAAAGGCACAGGCTGCGTCCTGCTG
V730C	XL-GB1-#35	GCCATCTGGCAGATTTGTGACCCCTTGCACCG	A834C	XL-GB1-#45	CAGGACGCAGCCTTTTGCTTTGCCTCTCTGGCC
	XL-GB1-#36	CGGTGCAAGGGGTCACAAATCTGCCAGATGGC		XL-GB1-#46	GGCCAGAGAGGCAAAGCAAAAGGCTGCGTCCTG
D731C	XL-GB1-#37	CATCTGGCAGATTGTGTGCCCCTTGCACCGAACC	S837C	XL-GB1-#47	GCCTTTGCCTTTGCCTGTCTGGCCATCGTGTTC
	XL-GB1-#38	GGTTCGGTGCAAGGGGCACACAATCTGCCAGATG		XL-GB1-#48	GAACACGATGGCCAGACAGGCAAAGGCAAAGGC
P732C	XL-GB1-#17	CTGGCAGATTGTGGACTGCTTGCACCGAACCATTG	L838C	XL-GB1-#75	CTTTGCCTTTGCCTCTTGTGCCATCGTGTTCTCTTC
	XL-GB1-#18	CAATGGTTCGGTGCAAGCAGTCCACAATCTGCCAG		XL-GB1-#76	GAAGAGAACACGATGGCACAAGAGGCAAAGGCAAAG
M765C	XL-GB1-#69	CTGCAGCTCCAAGAAGTGCAATACGTGGCTTGGC	A839C	XL-GB1-#77	GCCTTTGCCTCTCTGTGCATCGTGTTCTCTTCC
	XL-GB1-#70	GCCAAGCCACGTATTGCACTTCTTGGAGCTGCAG		XL-GB1-#78	GGAAGAGAACACGATGCACAGAGAGGCAAAGGC
N766C	XL-GB1-#11	CAGCTCCAAGAAGATGTGTACGTGGCTTGGCATTTTC			
	XL-GB1-#12	GAAAATGCCAAGCCACGTACACATCTTCTTGGAGCTG			

Supplementary Figure 13

Name and sequence of the primers used to construct the indicated GB1 mutants.

GABA$_{B2}$ mutations

Mutation	Primer name	Primer sequence	Mutation	Primer name	Primer sequence
**P479C**	XL-GB2-#01	CGGAAGATCTCGCTTTGTCTGTATAGCATCCTG	**T653C**	XL-GB2-#07	GAAAACACCCACATGTGCATCTGGCTTGGCATTG
	XL-GB2-#02	CAGGATGCTATACAGACAAAGCGAGATCTTCCG		XL-GB2-#08	CAATGCCAAGCCAGATGCACATGTGGGTGTTTTC
**A486C**	XL-GB2-#29	GTATAGCATCCTGTCCTGTCTCACCATCCTCGGC	**I654C**	G2-I654C-F	CACCCACATGACCTGCTGGCTTGGCATTG
	XL-GB2-#30	GCCGAGGATGGTGAGACAGGACAGGATGCTATAC		G2-I654C-R	CAATGCCAAGCCAGCAGGTCATGTGGGTG
**L487C**	XL-GB2-#31	GCATCCTGTCCGCTTGCACCATCCTCGGCATG	**W655C**	XL-GB2-#09	CACCCACATGACCATCTGTCTTGGCATTGTCTAC
	XL-GB2-#32	CATGCCGAGGATGGTGCAAGCGGACAGGATGC		XL-GB2-#10	GTAGACAATGCCAAGACAGATGGTCATGTGGGTG
**L490C**	XL-GB2-#33	GTCCGCTCTCACCATCTGCGGCATGATCATGGCC	**L656C**	XL-GB2-#11	CACATGACCATCTGGTGTGGCATTGTCTACGCC
	XL-GB2-#34	GGCCATGATCATGCCGCAGATGGTGAGAGCGGAC		XL-GB2-#12	GGCGTAGACAATGCCACACCAGATGGTCATGTG
**G491C**	RG2G491C-F	CTCTCACCATCCTCTGCATGATCATGGCC	**G657C**	G2-G657C-F	CATGACCATCTGGCTTTGCATTGTCTACGCCTAC
	RG2G491C-R	GGCCATGATCATGCAGAGGATGGTGAGAG		G2-G657C-R	GTAGGCGTAGACAATGCAAAGCCAGATGGTCATG
**M494C**	RG2M494C-F	CCTCGGCATGATCTGTGCCAGCGCCTTCC	**I658C**	XL-GB2-#43	GACCATCTGGCTTGGCTGTGTCTACGCCTACAAG
	RG2M494C-R	GGAAGGCGCTGGCACAGATCATGCCGAGG		XL-GB2-#44	CTTGTAGGCGTAGACACAGCCAAGCCAGATGGTC
**A497C**	RG2A497C-F	CATGATCATGGCCAGCTGCTTCCTCTTCTTCAAC	**V659C**	G2-V659C-F	CATCTGGCTTGGCATTTGCTACGCCTACAAGGG
	RG2A497C-R	GTTGAAGAAGAGGAAGCAGCTGGCCATGATCATG		G2-V659C-R	CCCTTGTAGGCGTAGCAAATGCCAAGCCAGATG
**L521C**	RG2L521C-F	CCTACATGAACAACTGCATCATCCTGGGAG	**Y660C**	XL-GB2-#45	CTGGCTTGGCATTGTCTGCGCCTACAAGGGGCTC
	RG2L521C-R	CTCCCAGGATGATGCAGTTGTTCATGTAGG		XL-GB2-#46	GAGCCCCTTGTAGGCGCAGACAATGCCAAGCCAG
**G525C**	RG2G525C-1F	CAACCTCATCATCCTGTGTGGAATGCTGTCCTATG	**A661C**	G2-A661C-F	CTTGGCATTGTCTACTGCTACAAGGGGCTCCTC
	RG2G525C-1R	CATAGGACAGCATTCCACACAGGATGATGAGGTTG		G2-A661C-R	GAGGAGCCCCTTGTAGCAGTAGACAATGCCAAG
**S532C**	RG2S532C-F	GCTGTCCTATGCATGCATCTTCCTCTTTG	**Y662C**	XL-GB2-#21	GGCATTGTCTACGCCTGCAAGGGGCTCCTCATG
	RG2S532C-R	CAAAGAGGAAGATGCATGCATAGGACAGC		XL-GB2-#22	CATGAGGAGCCCCTTGCAGGCGTAGACAATGCC
**F536C**	RG2F536C-F	CATCCATCTTCCTCTGTGGCCTCGATGGGTC	**K663C**	G2-K663C-F	CATTGTCTACGCCTACTGTGGGCTCCTCATGC
	RG2F536C-R	GACCCATCGAGGCCACAGAGGAAGATGGATG		G2-K663C-R	GCATGAGGAGCCCACAGTAGGCGTAGACAATG
**D539C**	RG2D539C-F	CTTCCTCTTTGGCCTCTGTGGGTCCTTCGTCTC	**G664C**	G2-G664C-F	GTCTACGCCTACAAGTGTCTCCTCATGCTATTC
	RG2D539C-R	GAGACGAAGGACCCACAGAGGCCAAAGAGGAAG		G2-G664C-R	GAATAGCATGAGGAGACACTTGTAGGCGTAGAC
**Q594C**	Sun-G2594-F	GAAGATCATCAAAGACTGTAAGCTGCTTGTGATTG	**L665C**	G2-L665C-F	CTACGCCTACAAGGGGTGCCTCATGCTATTCGG
	Sun-G2594-R	CAATCACAAGCAGCTTACAGTCTTTGATGATCTTC		G2-L665C-R	CCGAATAGCATGAGGCACCCCTTGTAGGCGTAG
**V598C**	Sun-G2598-F	GACCAGAAGCTGCTTTGCATTGTGGGGGCATG	**L666C**	XL-GB2-#47	GCCTACAAGGGGCTCTGCATGCTATTCGGTTG
	Sun-G2598-R	CATGCCCCCACAATGCAAAGCAGCTTCTGGTC		XL-GB2-#48	CAACCGAATAGCATGCAGAGCCCCTTGTAGGC
**G602C**	Sun-G2602-F	CTTGTGATTGTGGGGTGCATGCTGCTCATCGAC	**G698C**	Sun-G2698-F	CATGAGTGTGTACAATTGCGGGATCATGTGCATC
	Sun-G2602-R	GTCGATGAGCAGCATGCACCCCACAATCACAAG		Sun-G2698-R	GATGCACATGATCCCGCAATTGTACACACTCATG
**L605C**	Sun-G2605-F	GTGGGGGGCATGCTGTGCATCGACCTGGCCATC	**G699C**	G2-G698C-F	GAGTGTGTACAATGTGTGTATCATGTGCATCATC
	Sun-G2605-R	GATGGCCAGGTCGATGCACAGCATGCCCCCCAC		G2-G698C-R	GATGATGCACATGATACACACATTGTACACACTC
**I606C**	Sun-G2606-F	GGGGGCATGCTGCTCTGCGACCTGGCCATCCTG	**I700C**	G2-I699C-F	GTGTACAATGTGGGGTGCATGTGCATCATCGG
	Sun-G2606-R	CAGGATGGCCAGGTCGCAGAGCAGCATGCCCCC		G2-I699C-R	CCGATGATGCACATGCACCCCACATTGTACAC
**D607C**	Sun-G2607-F	GGCATGCTGCTCATCTGCCTGGCCATCCTGATC	**M701C**	G2-M700C-F	GTACAATGTGGGGATCTGTTGCATCATCGGGGCTG
	Sun-G2607-R	GATCAGGATGGCCAGGCAGATGAGCAGCATGCC		G2-M700C-R	CAGCCCCGATGATGCAACAGATCCCCACATTGTAC
**L608C**	Sun-G2608-F	CATGCTGCTCATCGACTGTGCCATCCTGATCGC	**I703C**	Sun-G2703-F	GTGGGGATCATGTGCTGCATCGGGGCTGCTGTC
	Sun-G2608-R	GCGATCAGGATGGCACAGTCGATGAGCAGCATG		Sun-G2703-R	GACAGCAGCCCCGATGCAGCACATGATCCCCAC
**C609A**	XL-GB2-#13	CTGCTCATCGACCTGGCCATCCTGATCTGTTGG	**I704C**	G2-I703C-F	GGGATCATGTGCATCTGCGGGGCTGCTGTCTC
	XL-GB2-#14	CCAACAGATCAGGATGGCCAGGTCGATGAGCAG		G2-I703C-R	GAGACAGCAGCCCCGCAGATGCACATGATCCC
**I610C**	Sun-G2610-F	CTCATCGACCTGGCCTGCCTGATCGCTTGGCAG	**G705C**	G2-G704C-F	GATCATGTGCATCATCTGTGCTGCTGTCTCCTTC
	Sun-G2610-R	CTGCCAAGCGATCAGGCAGGCCAGGTCGATGAG		G2-G704C-R	GAAGGAGACAGCAGCACAGATGATGCACATGATC
**L611C**	Sun-G2611-F	CATCGACCTGGCCATCTGTATCGCTTGGCAGGCTG	**A706C**	XL-GB2-#50	CATGTGCATCATCGGGTGTGCTGTCTCCTTCCTG
	Sun-G2611-R	CAGCCTGCCAAGCGATACAGATGGCCAGGTCGATG		XL-GB2-#51	CAGGAAGGAGACAGCACACCCGATGATGCACATG
**I612C**	Sun-G2612-F	GACCTGGCCATCCTGTGCGCTTGGCAGGCTGTG	**A707C**	XL-GB2-#23	GTGCATCATCGGGGCTTGTGTCTCCTTCCTGACAC
	Sun-G2612-R	CACAGCCTGCCAAGCGCACAGGATGGCCAGGTC		XL-GB2-#24	GTGTCAGGAAGGAGACACAAGCCCCGATGATGCAC
**C613A**	XL-GB2-#15	CTGTGCATCCTGATCGCTTGGCAGGCTGTGGAC	**V708C**	XL-GB2-#25	CATCATCGGGGCTGCTTGCTCCTTCCTGACACGTG
	XL-GB2-#16	GTCCACAGCCTGCCAAGCGATCAGGATGCACAG		XL-GB2-#26	CACGTGTCAGGAAGGAGCAAGCAGCCCCGATGATG
**W614C**	Sun-G2614-F	CATCCTGATCGCTTGTCAGGCTGTGGACCCC	**S709C**	XL-GB2-#53	CATCGGGGCTGCTGTCTGCTTCCTGACaCGTGAC
	Sun-G2614-R	GGGGTCCACAGCCTGACAAGCGATCAGGATG		XL-GB2-#54	GTCACGtGTCAGGAAGCAGACAGCAGCCCCGATG
**Q615C**	Sun-G2615-F	CATCCTGATCGCTTGGTGTGCTGTGGACCCCCTGC	**F710C**	XL-GB2-#55	GGGGCTGCTGTCTCCTGCCTGACaCGTGACCAG
	Sun-G2615-R	GCAGGGGGTCCACAGCACACCAAGCGATCAGGATG		XL-GB2-#56	CTGGTCACGtGTCAGGCAGGAGACAGCAGCCCC
**A616C**	Sun-G2616-F	CTGATCGCTTGGCAGTGTGTGGACCCCCTGCGG	**L711C**	XL-GB2-#27	GCTGCTGTCTCCTTCTGTACaCGTGACCAGCCC
	Sun-G2616-R	CCGCAGGGGGTCCACACACTGCCAAGCGATCAG		XL-GB2-#28	GGGCTGGTCACGtGTACAGAAGGAGACAGCAGC
**V617C**	Sun-G2617-F	GATCGCTTGGCAGGCTTGTGACCCCCTGCGGAGG	**F720C**	XL-GB2-#57	CAGCCCAACGTGCAGTGCTGCATCGTGGCCCTG
	Sun-G2617-R	CCTCCGCAGGGGGTCACAAGCCTGCCAAGCGATC		XL-GB2-#58	CAGGGCCACGATGCAGCACTGCACGTTGGGCTG
**D618C**	XL-GB2-#39	CTGTTGGCAGGCTGTGTGCCCCCTGCGGAGGACAG	**A724C**	XL-GB2-#59	CAGTTCTGCATCGTGTGCCTGGTCATCATCTTC
	XL-GB2-#40	CTGTCCTCCGCAGGGGGCACACAGCCTGCCAACAG		XL-GB2-#60	GAAGATGATGACCAGGCACACGATGCAGAACTG
**P619C**	XL-GB2-#19	GTTGGCAGGCTGTGGACTGCCTGCGGAGGACAGTAG	**L725C**	XL-GB2-#61	GTTCTGCATCGTGGCCTGTGTCATCATCTTCTGC
	XL-GB2-#20	CTACTGTCCTCCGCAGGCAGTCCACAGCCTGCCAAC		XL-GB2-#62	GCAGAAGATGATGACACAGGCCACGATGCAGAAC
**M652C**	XL-GB2-#41	CTGCGAAAACACCCACTGTACCATCTGGCTTGGC	**V726C**	RG2V726C-F	GCATCGTGGCCCTGTGCATCATCTTCTGCAG
	XL-GB2-#42	GCCAAGCCAGATGGTACAGTGGGTGTTTTCGCAG		RG2V726C-R	CTGCAGAAGATGATGCACAGGGCCACGATGC
			**C609A-C613A**	XL-GB2-#17	CTGGCCATCCTGATCGCTTGGCAGGCTGTGGAC
				XL-GB2-#18	GTCCACAGCCTGCCAAGCGATCAGGATGGCCAG

Supplementary Figure 14

Name and sequence of the primers used to construct the indicated GB2 mutants.
